# Prime time for chronic kidney disease

**DOI:** 10.1186/s12882-023-03340-w

**Published:** 2023-10-06

**Authors:** David S. Weisman, Sumeska Thavarajah, Bernard G. Jaar

**Affiliations:** 1 Department of Internal Medicine, MedStar Union Memorial Hospital, Baltimore, MD USA; 2 Department of Medicine, Georgetown University School of Medicine, Washington, DC USA; 3 Department of Medicine, Division of Nephrology, Johns Hopkins University School of Medicine, Baltimore, MD USA; 4 Department of Epidemiology, Johns Hopkins Bloomberg School of Public Health, Baltimore, MD USA; 5 The Welch Center for Prevention, Epidemiology, and Clinical Research, Johns Hopkins Medical Institutions, Baltimore, MD USA

**Keywords:** Chronic kidney disease, Primary care practitioners, Cardiovascular Disease, Mortality, Morbidity

## Abstract

Chronic kidney disease (CKD) represents a public health burden worldwide and is associated with significant morbidity and mortality. Most patients with CKD are managed by primary care practitioners and this educational series hope to improve knowledge and delivery of care to this high-risk patient population with CKD.

## Background

**Fig. 1:**
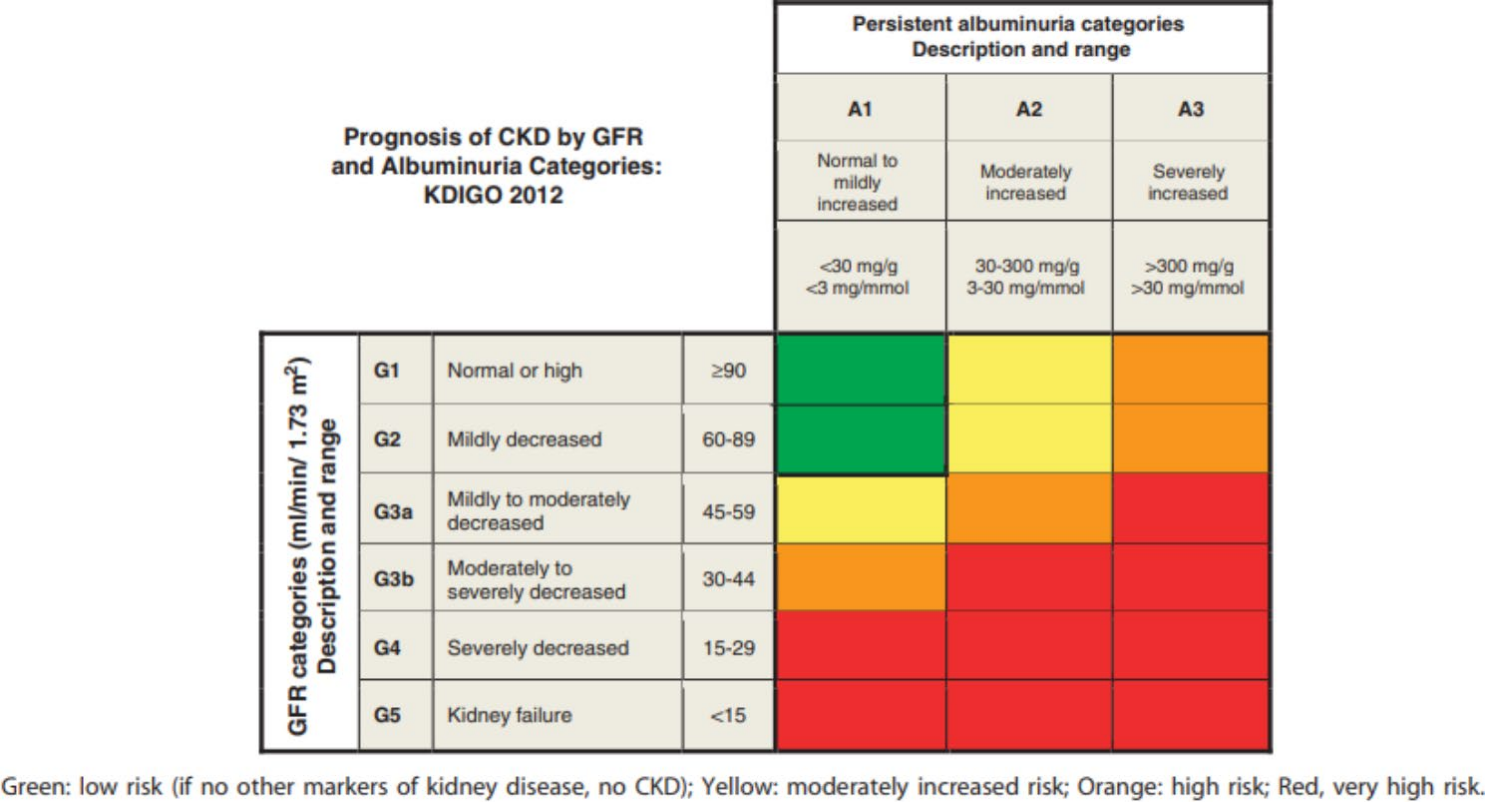
Reprinted with permission from KDIGO: Kidney Disease: Improving Global Outcomes (KDIGO) CKD Work Group. KDIGO 2012 clinical practice guideline for the evaluation and management of chronic kidney disease. Kidney Int Suppl. 2013;3:1-150. Copyright 2013

*BMC Nephrology* and *BMC Primary Care* are teaming up to bring a much-needed series for the primary care practitioner serving as an educational update of chronic kidney disease (CKD). This series will feature articles focused on the identification, diagnosis, management of complications, and treatment of patients with CKD geared towards the primary care practitioner perspective. CKD is a leading cause of medical illness worldwide with an estimated 8 to 16% of the world’s population and 15% of the US population affected [1, 2]. CKD is defined by a decrease in glomerular filtration rate (GFR) sustained over at least 3 months and then categorized in 5 stages based on GFR and 3 stages based on albuminuria, making up the CKD Heat Map (Fig. 1). Normal GFR is defined as greater or equal to 90 mL/min/1.73m^2^ and normal albuminuria is defined as urine albumin-to-creatinine ratio (ACR) < 30 mg/gram or < 3 mg/mmol. Decreasing GFR and increasing albuminuria define the various stages of CKD [3]. The prevalence increases with age and most patients are in CKD stage 3 at any one point in time [4]. The lifetime risk of developing CKD stage 3a in the United States is estimated to be greater than 50% [5].

CKD is associated with many biochemical and physiologic complications, most importantly being an independent risk factor for cardiovascular disease and mortality [6]. Patients at any stage of CKD are more likely to die of cardiovascular disease than require renal replacement therapy. The impact of COVID-19 was devasting on many patients with chronic medical conditions, particularly those with CKD. In a recent systematic review, the odds ratio of developing severe COVID-19 in patients with CKD was 2.1 (95% CI 1.2–3.8). The authors concluded in patients with CKD the risk of hospitalization is increased with COVID-19 compared to those without CKD. From their analysis the pooled mortality rate for patients with CKD stage 3 was 1.5 times higher (Hazard ratio 1.46, 95%CI 1.41–1.51) and 2.8 times higher for higher stages of CKD (harzard ratio 2.84, 95% 2.69–2.99) [7]. Primary care practitioners are uniquely positioned to take the lead not only in screening, diagnosis, and prevention of complications of CKD, but also to educate patients as to the necessary steps they can take to help slow progression of CKD.

## Risks and challenges with diagnosis

Although screening for CKD has not yet been advocated by The Unites States Preventative Services Task Force, screening is recommended for patients with increased risk by several kidney organizations, including the National Kidney Foundation in the United States [8]. Appropriate groups for screening include age over 60 years and older, presence of diabetes and hypertension, family history of diabetes, hypertension or kidney disease, and previous history of acute kidney injury and certain ethnic and racial minorities. There are numerous other causes of CKD including tubulointerstitial diseases, post-obstructive uropathies, cystic/genetic glomerular diseases, use of nephrotoxic medications such as nonsteroidal anti-inflammatory drugs (NSAIDs), obesity, and social determinants of health that impact access to care [9]. Kidney Disease: Improving Global Outcomes (KDIGO) guidelines recommend screening for proteinuria in all patients with CKD. Current trends in proteinuria screening in CKD have improved but continue to vary widely, noted to be between 10 and 45% in one large health care system in the US [10]. Using serum creatinine levels, a raceless calculation of estimated GFR based on the CKD-EPI Creatinine Eq. (2021) is recommended by the National Kidney Foundation [11]. The National Kidney Foundation and The American Society of Nephrology recently lead a task force to look at the formula to estimate GFR and have identified race as a social construct and not appropriate to be part of the estimated GFR equation. This can lead to overestimation of GFR in Blacks and result in delayed referral for kidney transplant evaluation or for referral to nephrology [12]. Again, the joint task force recommends use of the 2021 CKD Epidemiology Collaboration creatinine equation without race. The task force also recommends using cystatin C when appropriate or needed to accurately determine the estimated GFR [12]. Universal use of cystatin C was not recommended due to lack of availability in all healthcare settings and the prohibitive cost for large scale screenings. Measuring albuminuria via the ACR is appropriate to help classify the stages of CKD.

## Why does it matter?

CKD is associated with many complications in addition to the increased risk of cardiovascular disease. Cardiovascular complications in CKD are the leading cause of death in all stages of disease including transplant patients [6, 13]. Most CKD patients are more likely to die of cardiovascular complications than to start renal replacement therapy. The decline in GFR is linearly associated with increasing mortality, for example a GFR reduction of 40% is associated with an adjusted hazard ratio of 2.4; 95% CI (2.2–2.6) for death compared to a patient with a normal GFR [14]. Aggressive treatment of underlying risk factors is essential to manage cardiovascular burden of CKD including aggressive treatment of hypertension, lipid disorders, diabetes, and smoking cessation. Recognizing that social determinants of health such as socioeconomic status, specifically lower income status, by contributing to food insecurity and barriers to access to care, has been shown to have a direct effect on increased risk of CKD and of progression to end-stage kidney disease (ESKD) [15]. These social determinants of health may account in part for the differences in outcomes among different races. In 2020 the mortality rate among White patients with CKD was 9%, compared to 12% among Hispanics and 23% among Blacks [16]. There are several other complications of CKD that primary care physicians should be knowledgeable about including bone health, dysregulation between calcium and phosphate, anemia, hyperkalemia, and metabolic acidosis.

## Why primary care practitioners need to be involved?

Currently there are about 37 million US adults living with CKD, that’s more than 1 in 7 adults, with the majority in CKD stages 1 to 3 not being followed by a nephrologist (2). This trend may be worse in developing country (17). It is at the early stages of CKD that we can have the greatest impact on slowing progression of disease. For example, primary care practitioners make up the largest portion of the US providers work force and according to the Agency for Healthcare Research and Quality, are primed to meet the goals of the triple aim of US healthcare by improving quality of care, containing costs, and improving patient experience (18). Therefore, identifying those at risk or with CKD and preventing complications of CKD will be essential skills for the primary care practitioner with an aging patient population. In addition, there is a shortage of nephrologists in the workforce that has continued to decline and won’t be able to meet the needs of our patients with CKD [19]. Primary care practitioners must work alongside their nephrology colleagues and know the right time for referral. KDIGO guidelines recommend referral to a nephrologist when GFR falls below 30mL/min/1.73m^2^ or if urine ACR is greater than 300 mg/gram [3]. Simple steps can be taken to help patients with CKD improve their outcomes by educating them regarding their risk factors for CKD progression, on avoidance of nephrotoxins primarily NSAIDs and managing appropriate medication dosing such as antibiotics and oral anticoagulants [3]. Patients with CKD are also at increased risk of infections and primary care practitioners should be knowledgeable of current CDC vaccine recommendations [20]. Prognosis in CKD can be predicted at steady state with the use of the Kidney Failure Risk Equation (KFRE) developed and validated in many populations to determine both 2-year and 5-year risk of ESKD (https://kidneyfailurerisk.com). Optimal preparation for renal replacement therapy is essential to limit complications to patients, improve their sense of control, and to increase use of home dialysis modalities and potentially pre-emptive transplantation. Kidney transplants offer patients not only a better quality of life compared to dialysis but also a significantly extended life.

## Conclusions

Today is a very exciting time in nephrology and primary care as we now have many more tools in our armamentarium to significantly limit the risk of CKD progression to ESKD, reduce cardiovascular morbidity and improve overall survival, above and beyond the well-known renin-angiotensin-aldosterone system blockade agents. Several new medications such as the sodium–glucose cotransporter-2 inhibitors (SGLT2is) have been developed, proven in several multicenter international randomized clinical trials and approved by the Food and Drug Administration to benefit both diabetic and non-diabetic CKD patients. Similar benefits among diabetic CKD patients have been shown with the new nonsteroidal mineralocorticoid receptor antagonist, finerenone. This series will highlight the evidence and recent advances in nephrology to assist our primary care colleagues optimize the care of CKD patients, improve their quality of life and survival.

## Data Availability

Not applicable.

## List of abbreviations

ACR: albumin to creatinine ratio
CKD: chronic kidney disease
ESKD: end-stage kidney disease
GFR: glomerular filtration rate
KDIGO: Kidney Disease:Improving Global Outcomes
KFRE: Kidney Failure Risk Equation
NSAIDs: nonsteroidal anti-inflammatory drugs
SGLT2is: sodium–glucose cotransporter-2 inhibitors

## References

[1] Jha V, Garcia-Garcia G, Iseki K (2013). Chronic kidney disease: global dimension and perspectives. Lancet.

[2] Centers for Disease Control and Prevention (2021). Chronic kidney disease in the United States, 2021.

[3] Kidney Disease: Improving Global Outcomes (KDIGO) CKD Work Group (2013). KDIGO 2012 clinical practice guideline for the evaluation and management of chronic kidney disease. Kidney Int Suppl.

[4] Coresh J, Selvin E, Stevens LA (2007). Prevalence of chronic kidney disease in the United States. JAMA.

[5] Grams ME, Chow EK, Segev DL, Coresh J (2013). Lifetime incidence of CKD stages 3–5 in the United States. Am J Kidney Dis.

[6] Go AS, Chertow GM, Fan D, McCulloch CE, Hsu CY (2004). Chronic kidney disease and the risks of death, cardiovascular events, and hospitalization. N Engl J Med.

[7] Jdiaa SS, Mansour R, El Alayli A, Gautam A, Thomas P, Mustafa RA (2022). COVID-19 and chronic kidney disease: an updated overview of reviews. J Nephrol.

[8] Moyer VA, on behalf of the U.S. Preventative Services Task Force (2012). Screening for chronic kidney disease: U.S. Preventative Services Task Force Recommendation Statement. Ann Intern Med.

[9] Kazancioğlu R. Risk factors for chronic kidney disease: an update. Kidney Int Suppl (2011). 2013;3 (4):368–371. 10.1038/kisup.2013.7944. 10.1038/kisup.2013.79 PMC4089662 25019021

[10] Perkins RM, Chang AR, Wood KE, Coresh J, Matsushita K, Grams M (2016). Incident chronic kidney disease: trends in management and outcomes. Clin Kidney J.

[11] Kramer HJ, Jaar BG, Choi MJ, Palevsky PM, Vassalotti JA, Rocco MV, National Kidney Foundation Kidney Disease Outcomes Quality Initiative (2022). An endorsement of the removal of race from GFR estimation equations: a position Statement from the national kidney Foundation kidney Disease Outcomes Quality Initiative. m J Kidney Dis.

[12] Delgado C, Baweja M, Crews DC (2022). A Unifying Approach for GFR Estimation: recommendations of the NKF-ASN Task Force on reassessing the inclusion of race in diagnosing kidney disease. AJKD.

[13] Chen TK, Knicely DH, Grams ME (2019). Chronic kidney disease diagnosis and management: a review. JAMA.

[14] Coresh J, Turin TC, Matsushita K (2014). Decline in estimated glomerular filtration rate and subsequent risk of end-stage renal disease and mortality. JAMA.

[15] Banerjee T, Crews DC, Wesson DE, CDC CKD Surveillance Team (2017). Food insecurity, CKD, and subsequent ESRD in US adults. Am J Kidney Dis.

[16] United States Renal Data System. 2022 *USRDS Annual Data Report: Epidemiology of kidney disease in the United States* National Institutes of Health, National Institute of Diabetes and Digestive and Kidney Diseases, Bethesda, MD, 2022.

[17] Crews DC, Bello AK, Saadi G (2019). For theWorld kidney day Steering Committee. Burden, Access, and disparities in Kidnet Disease. Kidney Int.

[18] Primacre Care Owrkforce Facst and Stats. Conten last reviewed July 2018. Agency for Healthcare Research and Quality, Rockville, MD, https://www.ahrq.gov/research/findings/factsheets/primary/pcworkforce/index.html.

[19] Sharif MU, Elsayed ME, Stack AG (2016). The global nephrology workforce: emerging threats and potential solutions!. Clin Kidney J.

[20] Murthy N, Wodi AP, Bernstein H, Advisory Committee on Immunization Practices (2022). Recommended adult immunization schedule, United States, 2022. Ann Intern Med.

